# The New Disease, "Nona"

**Published:** 1890-04-12

**Authors:** 


### THE NEW DISEASE, "NONA."

On interviewing the Medical Superintendent of the Ospedale Maggiore at Milan, an institution with accommodation for from two to three thousand patients coming from all parts of Lombardy, a member of our staff learns that nothing is known of "Nona" there. No cases had been reported in the strictly medical journals, and no cases had been brought into hospital. The cases described in the papers were probably ordinary examples of trance presenting no features not already well understood in professional and scientific circles.

